# PM2.5 forecasting for an urban area based on deep learning and decomposition method

**DOI:** 10.1038/s41598-022-21769-1

**Published:** 2022-10-20

**Authors:** Nur’atiah Zaini, Lee Woen Ean, Ali Najah Ahmed, Marlinda Abdul Malek, Ming Fai Chow

**Affiliations:** 1 Institute of Sustainable Energy, Universiti Tenaga Nasional, 43000 Kajang, Selangor Malaysia; 2 Institute of Energy Infrastructure, Universiti Tenaga Nasional, 43000 Kajang, Selangor Malaysia; 3 Department of Civil Engineering, Kulliyyah of Engineering, International Islamic University Malaysia, 50728 Kuala Lumpur, Malaysia; 4 Discipline of Civil Engineering, School of Engineering, Monash Universiti Malaysia, 47500 Bandar Sunway, Selangor Malaysia

**Keywords:** Civil engineering, Environmental impact, Computational science

## Abstract

Rapid growth in industrialization and urbanization have resulted in high concentration of air pollutants in the environment and thus causing severe air pollution. Excessive emission of particulate matter to ambient air has negatively impacted the health and well-being of human society. Therefore, accurate forecasting of air pollutant concentration is crucial to mitigate the associated health risk. This study aims to predict the hourly PM2.5 concentration for an urban area in Malaysia using a hybrid deep learning model. Ensemble empirical mode decomposition (EEMD) was employed to decompose the original sequence data of particulate matter into several subseries. Long short-term memory (LSTM) was used to individually forecast the decomposed subseries considering the influence of air pollutant parameters for 1-h ahead forecasting. Then, the outputs of each forecast were aggregated to obtain the final forecasting of PM2.5 concentration. This study utilized two air quality datasets from two monitoring stations to validate the performance of proposed hybrid EEMD-LSTM model based on various data distributions. The spatial and temporal correlation for the proposed dataset were analysed to determine the significant input parameters for the forecasting model. The LSTM architecture consists of two LSTM layers and the data decomposition method is added in the data pre-processing stage to improve the forecasting accuracy. Finally, a comparison analysis was conducted to compare the performance of the proposed model with other deep learning models. The results illustrated that EEMD-LSTM yielded the highest accuracy results among other deep learning models, and the hybrid forecasting model was proved to have superior performance as compared to individual models.

## Introduction

High concentration of particulate matter in the ambient air has caused severe air pollution and other negative impacts in developing countries^1,2^. PM2.5 is a fine particle with a diameter of less than 2.5 µm, which recognize as one of the most dangerous pollutants that cause deterioration of air quality^3,4^. The inhalable particles of PM2.5 are commonly emitted from the combustion of solid and liquid fuels, domestic heating, and road vehicles. Therefore, the areas with a higher rate of industrial activities and traffic congestion are likely to have higher PM2.5 concentrations, which may also increase air pollution and harm human health. Besides that, long-term exposure to PM2.5 may lead to the increase of mortality risk due to respiratory and cardiovascular diseases^5^. Due to the vital effects of high PM2.5 concentration on the environment and human health, reliable forecasting of air pollutants has gained more attention recently to provide accurate information on air quality levels. Practical and precise forecasting of air quality is also essential to provide early warning to the public and enhance the decision-making process for necessary mitigation.

There are a lot of forecasting models that are developed based on time series analysis to forecast air pollutant concentration. The modelling approaches can be classified into three categories which are chemical transport models (CTM), statistical and artificial intelligence models^6^. Chemical transport models (CTM) predict air pollutants based on the transformation and chemical properties of the pollutants. The most common models for air quality forecasting are Community Multiscale Air Quality (CMAQ), Comprehensive Air Quality Model with Extensions (CAMx), Goddard Earth Observing System Atmospheric Chemistry (GEOS-Chem) and weather research forecasting (WRF). CTMs capable of dealing with the chemical reactions for air pollutant forecasting, however the models depend on various air pollutant data and the enormous amount of information for accurate forecasting makes it complicated. The models also operate based on extensive calculations that may limit the model performances^7,8^. Besides that, the statistical models such as autoregressive integrated moving average (ARIMA), grey model and regression models develop the statistical relationship between historical data of various influencing parameters with air pollutants. However, the statistical models exhibit limitations in learning large multidimensional and complex nonlinear time series data. The models are also unable to forecast multistep time horizons of the air pollutant based on numerous influencing variables^9^.

Considering the limitations of chemical transport and statistical models in learning and forecasting multistep ahead air pollutants based on various influencing parameters, artificial intelligence (AI) based technology such as machine learning and deep learning models have been established^6^. Machine learning models such as artificial neural network (ANN)^10,11^, support vector machine (SVM)^12^, extreme learning machine (ELM)^13^ and fuzzy logic^14^ with more sophisticated architectures are able to outperform the chemical transport and statistical models for air pollutant forecasting in terms of forecasting accuracy and time cost^15^. However, the techniques have the drawbacks of being limited in solving larger nonlinear time series datasets and incapable of efficiently capturing the features distribution of air quality datasets^16^. Deep learning is a new technology that has been globally applied to solve air quality forecasting problems and outweighs the performances of machine learning models due to its advantages in learning spatial and temporal distributions.

Understanding the importance of precise forecasting of air pollutant concentration has led to the increasing development of research and advanced forecasting models. In the last few years, deep learning has become a popular technique in the application of air quality forecasting and exhibits superior performance over the traditional neural network and other machine learning models^3,17,18^. Deep learning methods such as recurrent neural network (RNN), long short term memory (LSTM), convolutional neural network (CNN) and gated recurrent unit (GRU) are developed based on neural network architecture consisting of many processing layers. The methods are able to minimize the drawbacks of traditional neural networks in air quality time series problems and yield superior forecasting performances^19–21^. For instance, Ma et al.^22^ implemented a hybrid deep learning model based on LSTM for PM2.5 prediction. The study concludes that the proposed model outperformed other statistical and machine learning methods such as LASSO Regression, Ridge Regression, ANN, RNN and individual LSTM. Moreover, LSTM illustrates lowest forecasting error as compared to other individual and traditional machine learning models such as RNN, ANN and support vector regression (SVR). Besides that, Wang et al.^23^ summarized that the deep learning-based models such as GRU and LSTM can effectively forecast the real-time carbon monoxide concentration and yields better performance compared to nonlinear vector autoregression (VAR), radial basis functions network (RBFN) and SVM models. Comparing GRU and LSTM, it is found that LSTM performs slightly better compared to GRU. The results illustrate the reliability of the LSTM based model in solving nonlinear prediction problem.

Among the deep learning applications, it is learned that hybrid models have gained more interest in recent studies due to the advantages of enhancing prediction performance. Specifically, the combination of data decomposition based on empirical mode and deep learning techniques shows excellent forecasting performances and able to reduce the complexity of the dataset^1,24,25^. Huang et al.^25^ utilized empirical mode decomposition (EMD) to decompose the original PM2.5 sequence data and GRU to forecast the PM2.5 concentration. The ensemble model demonstrated high forecasting accuracy compared to other individual deep learning models such as LSTM, RNN and GRU. Although various meteorological parameters were considered to be influence variables in forecasting PM2.5 concentration, the study has neglected the effects of other air pollutant parameters on the forecasting. Besides that, GRU outperformed other individual models however, the method is based on simpler processing architecture units compared to LSTM. Therefore, LSTM may be an effective method in learning larger training datasets due to its advantage to memorize longer nonlinear sequence data. On the other hand, enhanced EMD, namely ensemble empirical mode decomposition (EEMD) with improved features, can eliminate the weakness in EMD and exhibit significant improvement for time series forecasting.

Bai et al.^24^ established an ensemble model of EEMD-LSTM to forecast hourly PM2.5 concentration at two air quality monitoring stations incorporating the meteorological parameters. The forecasting model showed superior performance compared to individual LSTM and feed-forward neural network (FFNN). However, this study also neglected the effect of other air pollutant parameters on forecasting and did not include the correlation analysis among input parameters that may effectively improve forecasting accuracy. Besides that, Ahani et al.^1^ applied EEMD to decompose original PM2.5 sequence data and LSTM is used as a forecasting tool based on five multistep ahead prediction strategies. Hybrid EEMD-LSTM based forecasting model illustrates good forecasting accuracy compared to individual LSTM. The results illustrate the effectiveness of decomposition method on the forecasting accuracy. However, it is found that EEMD-LSTM based model performs poorer as compared to EEMD-LSSVR based model for both shorter and longer forecasting horizons. Besides that, this study only considers PM2.5 concentration datasets collected from various air quality monitoring stations and neglects other influence parameters of air pollutant. This study also did not include spatial and temporal correlation analysis in selecting influence variables for LSTM model. Overall, the applications of hybrid data decomposition based on empirical mode and deep learning method are still very limited and extensive study is required for future model advancement.

Based on the abovementioned research, this study aims to forecast hourly PM2.5 concentration for an urban area using hybrid EEMD-LSTM considering the effects of other air pollutant parameters such as particulate matter (PM10), sulphur dioxide (SO_2_), nitrogen dioxide (NO_2_), ozone (O_3_) and carbon monoxide (CO). This study also proposed to validate the effectiveness of the hybrid model under different pollution levels using air quality datasets from two air quality monitoring stations. The main contributions of this study are: (i) consider the effects of other air pollutant parameters on PM2.5 forecasting, (ii) determine the correlations among the proposed features to identify the significant input variables to the forecasting model and conduct temporal correlation analysis based on autocorrelation function (ACF) to determine the historical input. Different monitoring locations may have different set of input variables and number of historical time step for optimum forecasting accuracy, (iii) EEMD is used to decomposed original PM2.5 sequence data due to its advantages over simple EMD, (iv) stacked LSTM architecture is established to individually train and forecast PM2.5 concentration at different locations. The individual forecasting output of LSTM models are aggregated to obtain the final forecasting, (v) forecasting performance of the proposed hybrid EEMD-LSTM is compared to other developed individual and hybrid deep learning based models such as LSTM, Bidirectional LSTM, EMD-LSTM, EMD-GRU and CNN-LSTM in order to investigate the model’s efficiency. The proposed forecasting model effectively forecasts PM2.5 concentration for 1-hour ahead of forecasting horizon based on the past hours and other influence parameters. The experimental results demonstrate that the proposed model has successfully forecasted PM2.5 concentration with excellent forecasting accuracy and outperformed other deep learning models in terms of four statistical evaluations. The improved method of EEMD also shows decent performance in decomposing complex time-series data to enhance the precision of the forecasting model.

## Data and methods

### Study area and data

**Figure 1:**
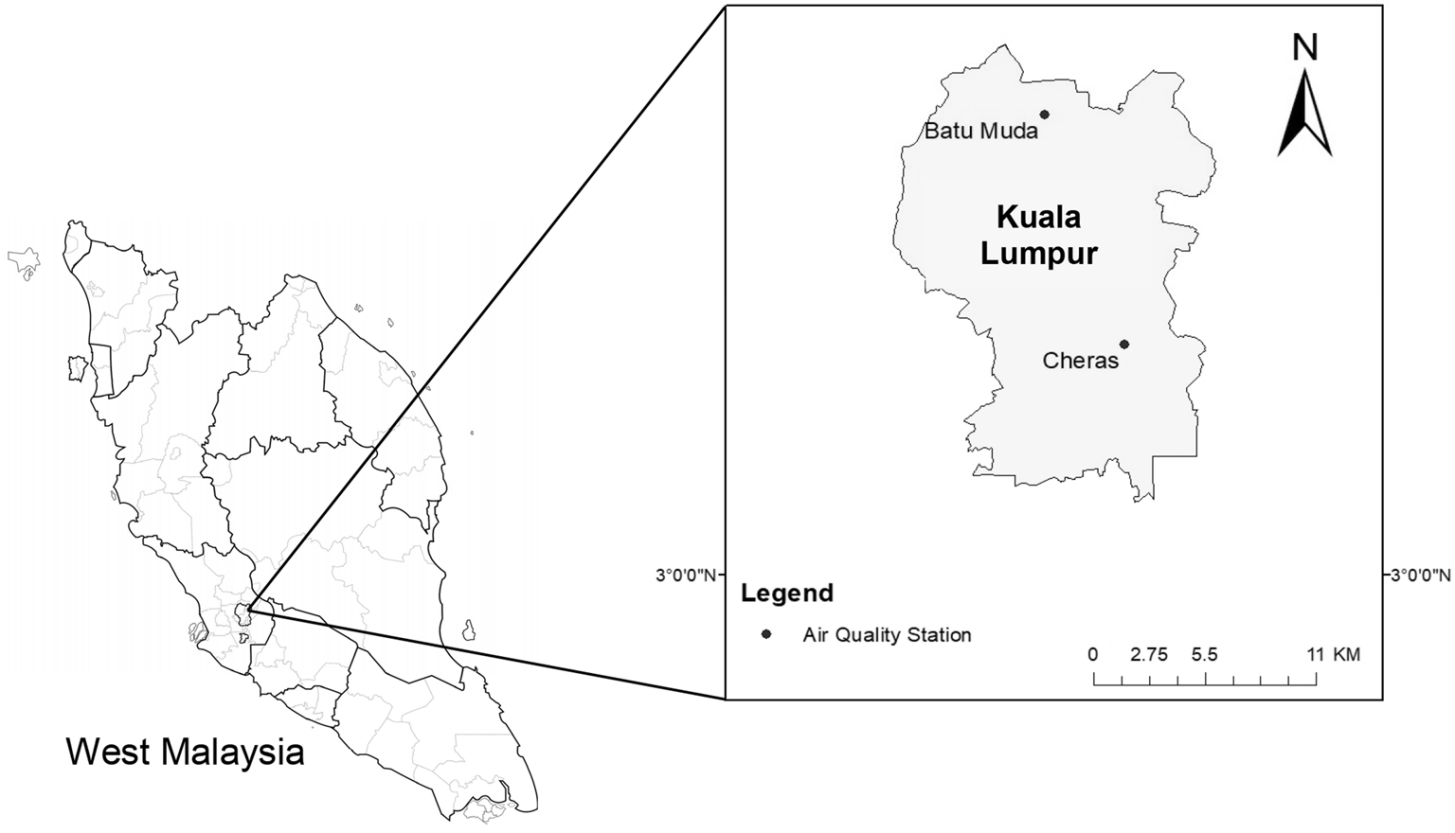
Air quality monitoring stations^40^.

This study utilizes hourly historical air quality dataset which consisting of six air pollutant parameters namely particulate matter (PM2.5 and PM10), sulphur dioxide (SO_2_), nitrogen dioxide (NO_2_), ozone (O_3_) and carbon monoxide (CO) from two air quality monitoring stations located in Kuala Lumpur, Malaysia. Kuala Lumpur is Malaysia’s capital city, the country’s most developed and densely populated city^26^. The reason for selecting such datasets is because Kuala Lumpur the capital city of Malaysia with the highest rate of industrial activities, urbanization and traffic congestion. This situation could contribute to higher air pollutants emission in the area. The datasets for both monitoring stations namely Cheras and Batu Muda are collected from Malaysia’s department of environment (DoE) during the period of 1 January 2018 to 31 December 2019. Figure 1 demonstrates the location of air quality monitoring stations within the selected study area. The time series datasets of both monitoring stations have a total of 17,520 data and are divided into two different datasets for subsequent forecasting. 70% of the total data was used to train the proposed model parameters while the remaining 30% was used to forecast the air pollutant concentration. Table 1 presents the descriptions of air pollutant parameters for Cheras and Batu Muda stations. In the data pre-processing process, the missing values were analyzed and encoded using the linear interpolation method. Lastly, the training and testing datasets were normalized in the range of [0,1] to prevent the non-uniform value used for accurate forecasting. The equation for data normalization is defined in Eq. (1).

1 $$z=\frac{x-\mathrm{min}(x)}{\mathrm{max}\left(x\right)-\mathrm{min}(x)},$$

where *z* is the normalized values and *x* is the observed values.

**Table 1:** Description of datasets used.

		PM10	PM2.5	SO2	NO2	O3	CO
Cheras	Max	291.8160	273.3470	0.0137	0.0664	0.1308	3.3010
	Min	2.5060	0.0710	0.0000	0.0001	0.0000	0.0790
	Mean	34.7517	25.5711	0.0009	0.0176	0.0218	0.8399
	Std dev	20.0555	18.0789	0.0008	0.0094	0.0226	0.3921
	Total no	17,520	17,520	17,520	17,520	17,520	17,520
Batu Muda	Max	283.1260	263.7520	0.0171	0.0635	0.1377	4.9140
	Min	0.0000	0.0000	0.0000	0.0001	0.0000	0.0400
	Mean	32.1283	24.8587	0.0010	0.0173	0.0157	0.9730
	Std dev	20.5827	18.4417	0.0007	0.0086	0.0180	0.3930
	Total no	17,520	17,520	17,520	17,520	17,520	17,520

### EEMD-LSTM architecture

This study proposed the application of ensemble empirical mode decomposition (EEMD) in data processing for time series forecasting using LSTM model. EEMD is an improved method of empirical mode decomposition (EMD) that has advantages over EMD. EMD with a simpler decomposition method is capable to extract the feature’s frequency without pre-determined basic functions. The technique is designed to discrete the complex time series into a simple oscillatory mode based on a local time scale. The separated mode is known as intrinsic mode functions (IMFs)^27,28^. However, EMD suffers from limitations of mode mixing which the condition of either a single IMF component consists of a different signal scale or a similar signal scale in different IMF components. Therefore, EEMD that adds white noise series in the targeted data is introduced to tackle the disadvantage of EMD in order to improve the decomposition performances^29^. EEMD decomposes the original PM2.5 concentration sequence data into several subsequences in the data processing stage for successive forecasting using LSTM.

LSTM is a variation of Recurrent Neural Network (RNN) that is able to deal with vanishing gradient problems. LSTM is found to remember both long-term and short-term series of values due to the advantages of special units’ architecture called memory block^30,31^. Moreover, LSTM consists of three gate units namely the input gate, forget, and output gates aim to control the movement of information and allow the network to learn recurrently^32–34^. In this study, stacked LSTM is used to individually forecast the decomposed subsequence before the final forecasting is obtained by aggregating the output values.

**Figure 2:**
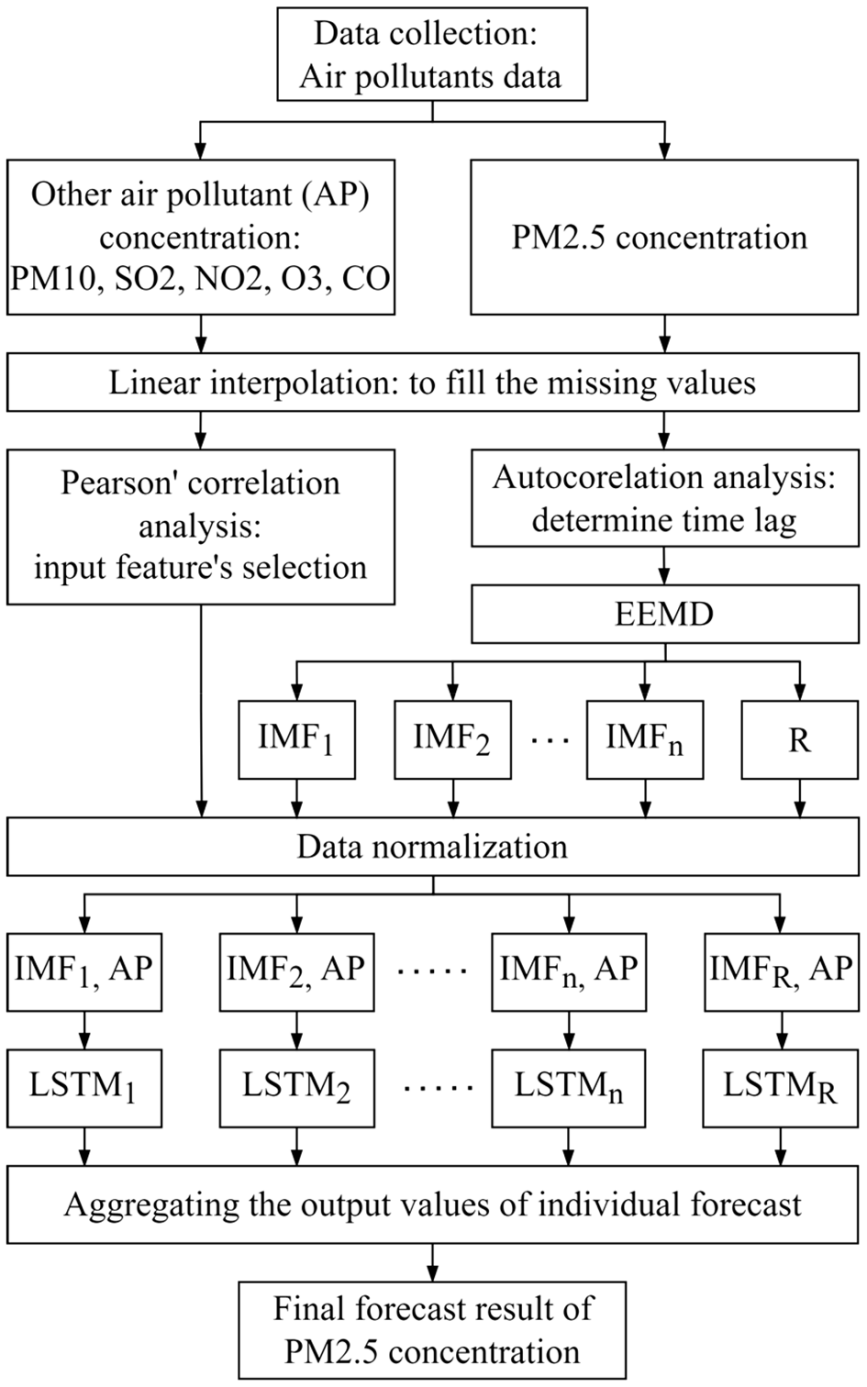
Procedure of EEMD-LSTM.

The hybrid EEMD-LSTM forecasting model consists of several modelling procedures, as illustrated in Fig. 2. The procedures can be summarized as follows.

- Collection of hourly historical data of air pollutants at two air quality monitoring stations. Two different datasets were collected for the forecasting model's validation purposes.
- In the data pre-processing stage, the datasets were analysed for missing values and the linear interpolation method is employed to fill the missing values.
- Analyse the influences of other air pollutant parameters on the changes of PM2.5 concentration values using Pearson’s correlation. The analysis is to identify the significant input parameters to the forecasting model for improving the forecasting performance. Besides that, determine the model’s historical input for the forecasting based on autocorrelation analysis. The input parameters and historical lag time of the proposed model may differ for different air quality monitoring stations.
- Perform EEMD to decompose nonlinear and complex PM2.5 concentration data into several subseries called IMFs and a residual.
- Construct separate stacked LSTM for multistep forecasting and determine the best-fit hyperparameters for the model. The value of hyperparameters is determined by continuously adjusting the values until the optimum performance is achieved. The input parameters for the forecasting models are the normalized data of decomposed PM2.5 and other air pollutant parameters.
- Aggregate the sequences of forecasted values from LSTM output to obtain the final forecasting of PM2.5 concentration. Compare the forecasted values to the observed values and evaluate the forecasting performances using four evaluation equations.

### Performance evaluation

The performance of the proposed LSTM-based model is evaluated using four different indicators namely root mean square error (RMSE), mean absolute error (MAE), mean absolute percentage error (MAPE) and coefficient of determination (R^2^). RMSE calculates the difference between forecasted and observed values at different time scales. MAE indicates the absolute difference between forecasted and observed values on overall data points. MAPE measures the forecasting accuracy based on the average absolute error of forecasted and observed values in terms of percentage. The lower value of RMSE, MAE and MAPE illustrates better forecasting performance. Meanwhile, R^2^ indicates the effect of the difference in observed values on the variation in forecasted values. The high value of R^2^ reflects the better performance of the forecasting model.

The statistical evaluations are defined based on the following equations.

2 $$RMSE=\sqrt{\frac{1}{n}\sum_{i=1}^{n}{\left({y}_{i}-{\widehat{y}}_{i}\right)}^{2}},$$

3 $$MAE=\frac{1}{n}\sum_{i=1}^{n}\left|{y}_{i}-{\widehat{y}}_{i}\right|,$$

4 $$MAPE=\frac{1}{n}\sum_{i=1}^{n}\left|\frac{{y}_{i}-{\widehat{y}}_{i}}{{y}_{i}}\right|\times 100,$$

5 $${R}^{2}=\frac{{\left[\sum_{i=1}^{n}({y}_{i}-{y}_{avg})({\widehat{y}}_{i}-{\widehat{y}}_{avg})\right]}^{2}}{{\sum_{i=1}^{n}({y}_{i}-{y}_{avg})}^{2}\times {\sum }_{i=1}^{n}{({\widehat{y}}_{i}-{\widehat{y}}_{avg})}^{2}},$$

where *n* is the number of data points. ${y}_{i}$ and ${\widehat{y}}_{i}$ are the observed and forecasted values of PM2.5 concentration, respectively. Meanwhile, ${\widehat{y}}_{avg}$ and ${y}_{avg}$ are the average of the actual and forecasted value of PM2.5 concentration.

## Experimental setup

### Features correlation

PM2.5 concentration within the study area could be affected by the emission of other air pollutants such as PM10, SO_2_, NO_2_, O_2_, O_3_. Therefore, the correlation between PM2.5 and other influenced air pollutant parameters was analysed in order to determine the influencing variables of PM2.5 concentration^25^. This study proposed Pearson’s correlation coefficient to evaluate the relationship between PM2.5 concentration and the influencing parameters. Pearson’s correlation can be defined as in Eq. (6).

6 $$r= \frac{\sum_{t=1}^{n}({x}_{t}-{\overline{x} }_{t})({y}_{t}-{\overline{y} }_{t})}{\sqrt{\sum_{t=1}^{n}{({x}_{t}-{\overline{x} }_{t})}^{2}\times \sum_{t=1}^{n}{({y}_{t}-{\overline{y} }_{t})}^{2}}},$$

where *n* is the number of observations in the dataset. *X*_*t*_ and *y*_*t*_ are historical PM2.5 concentrations and other air pollutants series, respectively. ${\overline{x} }_{t}$ and ${\overline{y} }_{t}$ are the mean value of historical PM2.5 concentration and other air pollutants series, respectively.

**Figure 3:**
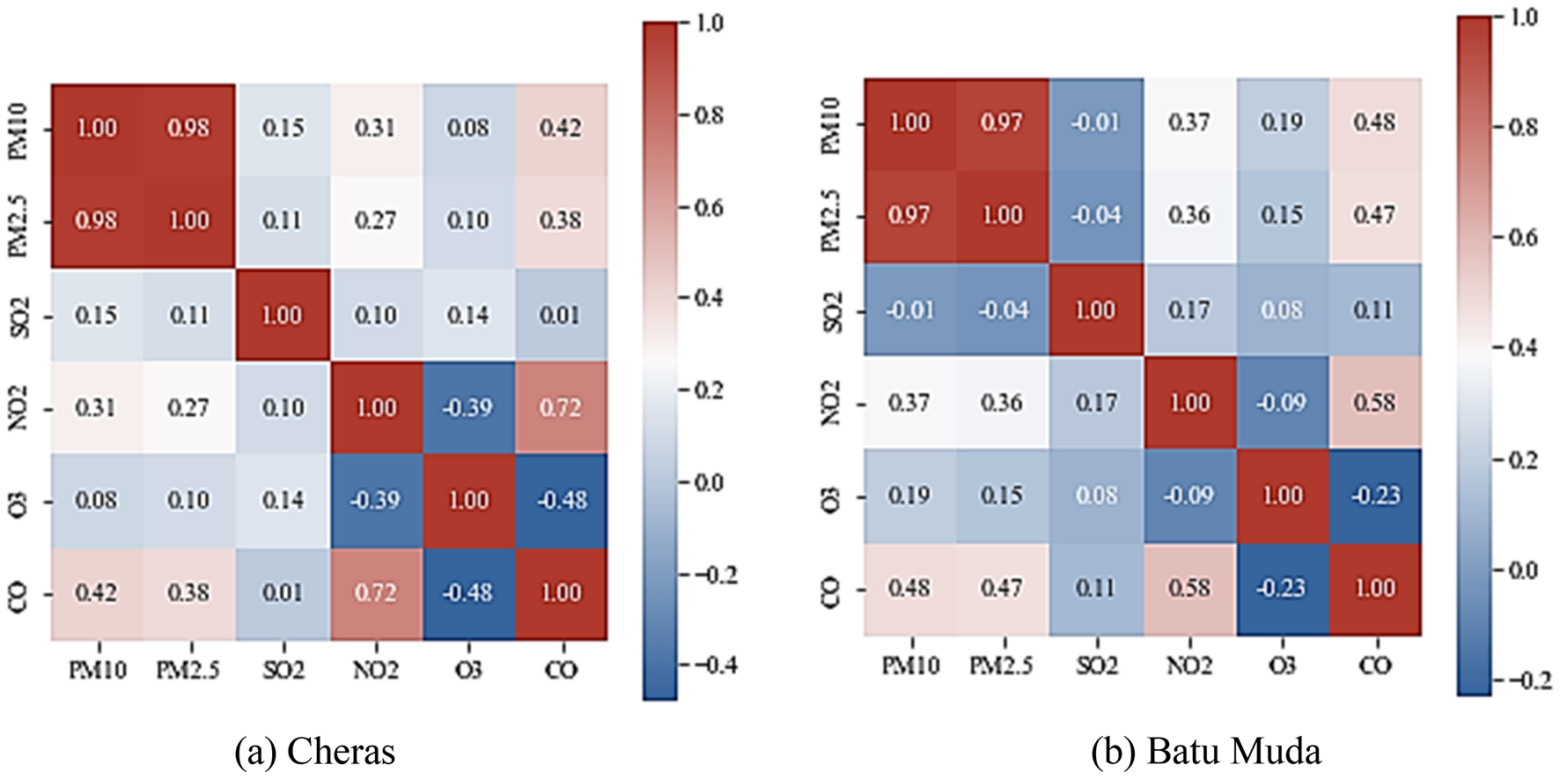
Pearson’s correlation of PM2.5 concentration and other features for (**a**) Cheras and (**b**) Batu Muda station.

The heatmap in Fig. 3 illustrates the correlation between PM2.5 and other air pollutants at both air quality monitoring stations within the study area. For Batu Muda station, PM10 has the highest correlation value of 0.97, indicating that the variable significantly influences PM2.5 concentration. Besides that, CO, O_3_ and NO_2_ also affect the changes in PM2.5 concentration. Meanwhile, the correlation value of SO_2_ is 0.04, which is closest to zero, indicating that the variable has the weakest correlation to PM2.5 concentration. Therefore, the input variables to the proposed forecasting model for Batu Muda station is designed without SO_2_ concentration. Similarly, PM10 at Cheras station has the highest correlation to PM2.5 concentration with a correlation value of 0.98. Other air pollutants also show a critical role in affecting the PM2.5 concentration values. Therefore, this study decided to select all influencing parameters as input variables to the proposed model to forecast PM2.5 concentration at Cheras station.

### Temporal correlation

For temporal analysis of PM2.5 concentration, the autocorrelation function (ACF) is used to analyse the correlation between time series data of different periods. The autocorrelation analysis may benefit the selection of time lag for historical input features to the proposed deep learning forecasting model^35^. For time delay *k*, the autocorrelation coefficient can be calculated as in Eq. (7).

7 $${r}_{k}=\frac{{\sum }_{i=1}^{n-k}({x}_{i}-\overline{x })({x}_{i+k}-\overline{x })}{{\sum }_{i=1}^{n}{({x}_{i}-\overline{x })}^{2}},$$

where ${x}_{i}$ and ${x}_{i+k}$ denote the sample value at time *i* and *i* + *k*, respective. Meanwhile, $\overline{x }$ is the sample mean of the sequence.

**Figure 4:**
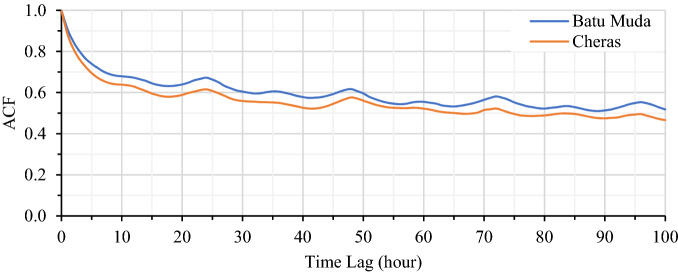
Autocorrelation coefficient of PM2.5 concentration.

The autocorrelation coefficient of time series air quality data for Cheras and Batu Muda stations is illustrated in Fig. 4. Overall, it can be perceived that the autocorrelation coefficient is decreases as time lag increases. It is indicated that the earlier data has an insignificant effect on the current air quality data^35^. Besides that, both stations recorded an autocorrelation coefficient of more than 0.5 at a time lag of 65 h. Therefore, the proposed model is trained using the selected time lag based on the performance analysis. The optimum time lag for historical input is significant to ensure the model is able to capture long-term sequence information for the next hour of forecasting. However, increasing time lag may lead to large dimensionality distribution and the model become unnecessarily complex. Besides that, the model will suffer from overfitting as well as reduce the forecasting performances. Hence, the optimal time lag for the model’s input is selected based on the evaluation of different hours.

The selection of time lag for the deep learning model is conducted based on a grid search assignment where several hour time lags are preselected ranging from 1 to 12 h. Small time lag produces unsatisfactory performance due to insufficient long-term memory input to the model. However, a large time lag may promote unnecessary inputs to the model and increase the model’s complexity. Therefore, the optimum time lag for historical input data is determined by analysing the performances of the deep learning model to forecast PM2.5 concentration. In this study, EEMD-LSTM is analysed for different time lags for 1-h interval for both Cheras and Batu Muda datasets. Table 2 shows the RMSE and R^2^ of the proposed model at multiple time lags for PM2.5 concentration forecasting. Both datasets have different performances based on the time lag analysis. It is found that, EEMD-LSTM performs the best at 6-h and 2-h time lag at Cheras and Batu Muda monitoring stations, respectively. The results presented in Fig. 4 can be explained that different datasets might have different autocorrelation values in the same time lag due to the distribution of data series. Therefore, the historical input for EEMD-LSTM model is set to 6 h for Cheras dataset and 2 h for the Batu Muda dataset to forecast 1-h PM2.5 concentration.

**Table 2:** Evaluation of EEMD-LSTM at different time lags for Cheras and Batu Muda datasets.

Lag time (h)	Cheras		Batu Muda	
	RMSE	R^2^	RMSE	R^2^
1	8.7932	0.8886	6.8829	0.9349
2	4.6773	0.9685	**4.8949**	**0.9673**
3	4.9216	0.9651	5.4320	0.9595
4	4.4857	0.9710	7.0627	0.9315
5	5.4730	0.9569	8.1152	0.9095
6	**4.2083**	**0.9780**	6.2291	0.9467
7	6.9881	0.9297	5.8072	0.9537
8	4.8443	0.9662	5.9531	0.9513
9	7.4410	0.9203	5.1341	0.9638
10	5.0353	0.9635	6.5568	0.9410
11	7.9558	0.9089	5.1510	0.9636
12	5.4208	0.9577	8.2213	0.9072

Significant values are in bold.

### Model’s architecture design

**Figure 5:**
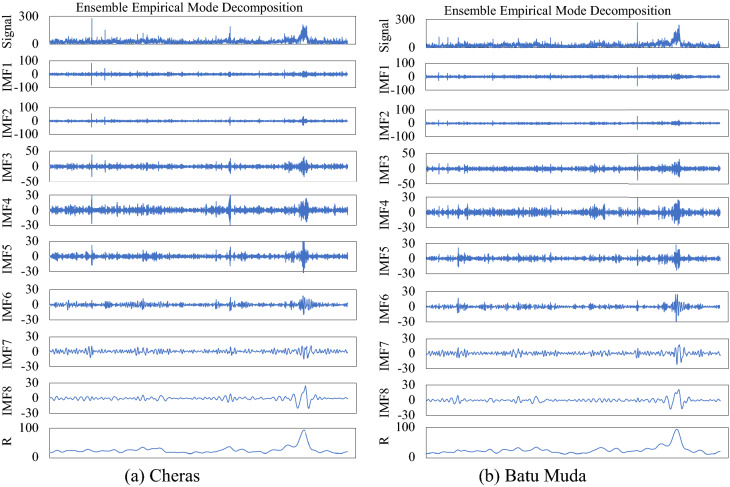
IMFs and residual plot of decomposed concentration data for (**a**) Cheras and (**b**) Batu Muda station.

This study proposes to focus on forecasting PM2.5 concentration using ensemble LSTM based on mode decomposition. Due to the nonlinearity and complexity of hourly time series PM2.5 concentration and the influences of other air pollutants, the data decomposition method based on empirical mode namely EEMD is proposed to improve the forecasting accuracy of LSTM based model. PM2.5 concentrations for Cheras and Batu Muda stations are decomposed into eight stationary subsequences called intrinsic mode function (IMFs) and a residue (R) in the data processing stage. Figure 5 represents the summary of decomposed time series data obtained for Cheras and Batu Muda stations. Every subsequence of decomposed PM2.5 is considered the independent dataset for the input to LSTM model. Nine LSTM models are separately developed to learn and forecast every decomposed sequence before integrating all forecasting outputs to obtain the final forecasting of the PM2.5 concentration value.

LSTM-based model is constructed based on stacked two LSTM layers with 128 hidden neurons in each layer. Other model’s hyperparameters are also determined, such as optimizer, learning rate, activation function and the number of epochs. A manual search is performed to find the optimum hyperparameter's values by continuously adjusting the values until the model reaches the best forecasting performance. Table 3 lists the parameters of the LSTM model for forecasting PM2.5 concentration. One of the main hyperparameters in the deep learning model is the optimizer. This study uses adaptive moment estimation (ADAM) as an optimization function that can successfully work in online and stationary settings as well as show better performance with sparse gradients. The exponential decay rate for first-moment estimates is set to 0.9 and the exponential decay rate for second-moment estimates is set to 0.999. Besides that, the activation function used in the network is rectified linear unit (ReLU), which can reduce the vanishing gradient and has better convergence performance. The forecasting model is fitted for a batch size of 128 and mean square error (MSE) is used as the loss function. The dropout rate for the forecasting models is set to 0.1 in order to avoid overfitting problems during the model's training. Early stopping criteria are used for stopping the training progress when the evaluation metric does not improve. The training epoch is initialized for 100 epochs. Moreover, the callbacks function of ReduceLROnPlateau is used to reduce the learning rate for enhancing the model's performance if the evaluation metric stops improving. The minimum limit of the learning rate is set to 0.00001. After the model has been successfully trained, the testing dataset is used to obtain the forecasting values of the sample sequences. Then, all forecasted subsequences are aggregated for final forecasting. Lastly, the forecasting model's performances are evaluated in terms of RMSE, MAE, MAPE and R^2^.

**Table 3:** Parameter setting for EEMD-LSTM.

Modelling strategy	Parameter name	Description
EEMD	Number of IMF	8
	Amplitude of the added noise	0.2
LSTM	Optimizer	Adam
	Number of LSTM unit	128, 128
	Learning rate	0.00001
	β_1_,β_2_	0.9, 0.999
	Activation function	ReLU
	Number of epochs	100
	Batch size	128
	Dropout	0.1
	Loss function	MSE

## Result and discussion

### Results of EEMD-LSTM

This study applies an ensemble model of EEMD-LSTM for forecasting PM2.5 concentration at two air quality monitoring stations in an urban area. The forecasting is performed by considering the effects of other air pollutants emissions at the respective monitoring station. EEMD is used to decompose the time series of PM2.5 concentration data into eight subsequences and a residue is used to reduce the complexity of time series data for accurate forecasting. Nine LSTM models are separately established for every independent decomposed subsequence. Forecasting output from each model is aggregated in order to obtain the final forecasting of PM2.5 concentration. Then, the performance of the proposed model is evaluated based on the statistical equations.

**Figure 6:**
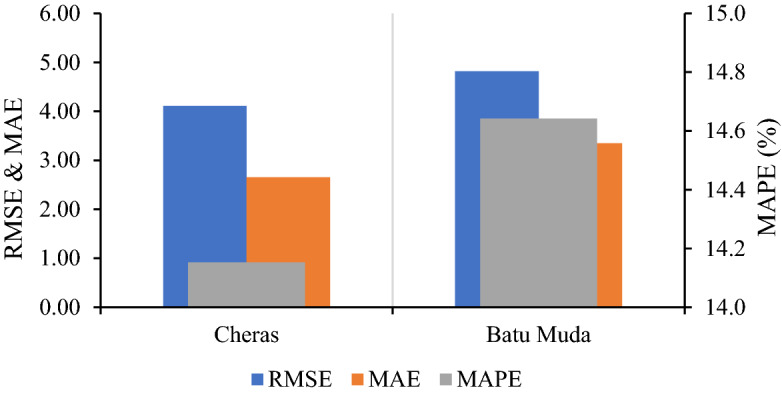
Evaluation error of EEMD-LSTM for Cheras and Batu Muda station.

**Figure 7:**
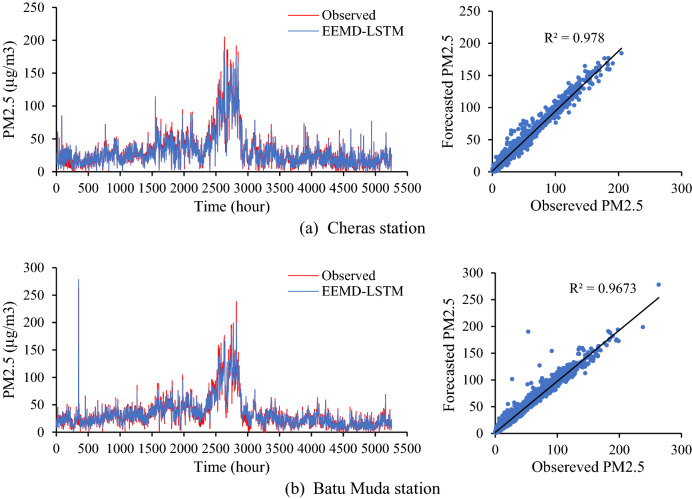
PM2.5 forecasting based on EEMD-LSTM for (**a**) Cheras (**b**) Batu Muda station.

The developed hybrid EEMD-LSTM model at both monitoring stations has the same architecture and experimental setup in order to investigate the model’s validity in learning and forecasting different time-series datasets. Due to the distribution of the air quality data, this study decided to set the model’s historical input based on time lag analysis by considering the effect of temporal correlation within the data series. Besides that, for the corresponding monitoring stations, the proposed forecasting model is set to different input parameters based on features correlation analysis which only the parameters with a high correlation value to the target variable are selected. EEMD-LSTM model at Cheras station with all air pollutant parameters as input variable and historical input of six-hour yield RMSE = 4.2083 μg/m^3^, MAE = 2.8190 μg/m^3^ and MAPE = 14.152%. Meanwhile, EEMD-LSTM model without SO_2_ concentration in the input sequence and two-hour historical input for Batu Muda station yields RMSE = 4.8949 μg/m^3^, MAE = 2.7724 μg/m^3^ and MAPE = 14.642%. Figure 6 summarizes the evaluation error of the EEMD-LSTM models at both monitoring stations. Final forecasting of one hour ahead and the distribution between forecasted and observed PM2.5 concentrations for the testing dataset at both air quality monitoring stations based on the respective input variables and historical time lags are presented in Fig. 7. The figure demonstrates the final forecasting follows the trend of actual values. Besides that, the distribution of both values converges to the centre crosswise of the graph approximately, demonstrating the higher accuracy of forecasting in terms of statistical evaluations. The evaluation results illustrate that the proposed forecasting model is able to forecast PM2.5 concentration with small errors and high accuracy for different datasets in an urban area. The improved decomposition method of EEMD has successfully decomposed and extracted the important characteristic of the complex time-series datasets to help in enhancing forecasting accuracy. Additionally, LSTM is able to learn and forecast large nonlinear and long-term dependence of PM2.5 concentration time series.

### Comparison study

Seven deep learning based models are established as benchmark models and compared to the proposed forecasting model. The comparative analysis aims to verify the efficiency of the proposed EEEMD-LSTM model. The comparative models namely EMD-LSTM, EMD-GRU, LSTM, Bidirectional LSTM, sequence to sequence LSTM, CNN-LSTM and GRU are built using the same parameters as the proposed model. All experiments utilizing both air quality datasets are conducted under a similar experimental setup to ensure the consistency of comparative analysis. The hyperparameter setting and descriptions of the comparative model are presented in Table 4. Meanwhile, Table 5 lists the forecasting performances of all comparative models and the proposed model in terms of RMSE, MAE, MAPE and R^2^ for both Cheras and Batu Muda stations. EEMD-LSTM yields the lowest forecasting errors and highest R^2^ as compared to the other seven deep learning models for both monitoring stations. The results prove that EEMD-LSTM is able to forecast PM2.5 concentration with high forecasting accuracy among the deep learning models.

**Table 4:** Description of comparative models.

Model	Description
EMD-LSTM	EMD = 8 IMFs, 1 Residual LSTM parameters as in Table 3
EMD-GRU	EMD = 8 IMFs, 1 Residual 2 GRU layer, number of nodes = 128, 128
LSTM	Table 3
Bi-LSTM	1 BiLSTM layer; number of nodes = 128 LSTM parameters as in Table 3
Seq2seq LSTM	Encoder-decoder model with 2 LSTM layers LSTM parameters as in Table 3
CNN-LSTM	Conv1D: filter = 5, kernel = 1; pooling size = 1 LSTM parameters as in Table 3
GRU	2 layers of GRU; number of nodes = 128, 128 Parameters setting same as in Table 3

**Table 5:** Forecasting evaluation of deep learning models.

	Cheras				Batu Muda			
	RMSE (μg/m^3^)	MAE (μg/m^3^)	MAPE (%)	R^2^	RMSE (μg/m^3^)	MAE (μg/m^3^)	MAPE (%)	R^2^
EEMD-LSTM	4.2083	2.8190	14.152	0.9780	4.8949	2.7724	14.642	0.9673
EMD-LSTM	8.3323	5.3211	27.189	0.8998	6.7878	4.5899	23.423	0.9366
EMD-GRU^25^	6.6668	4.3390	21.975	0.9359	6.6190	4.5921	23.580	0.9397
LSTM^36^	10.3188	6.6597	39.661	0.8464	10.3020	6.2035	33.960	0.8540
Bi-LSTM	10.1013	6.5553	36.564	0.8528	9.8595	6.2443	35.314	0.8663
Seq2seq LSTM	11.2707	7.1170	36.765	0.8167	12.1296	7.2301	32.980	0.7976
CNN-LSTM	12.0066	7.4250	38.480	0.7920	12.3783	7.3813	33.883	0.7893
GRU^37^	10.1057	6.5547	37.739	0.8526	11.9297	7.7404	34.471	0.8043

EEMD-LSTM decreases the forecasting error of EMD-LSTM by 49.49%, 47.02% and 47.95% for Cheras station, while 27.89%, 39.60% and 37.49% for Batu Muda station in terms of RMSE, MAE and MAPE, respectively. Besides that, EEMD-LSTM enhances the accuracy of EMD-LSTM in terms of R^2^ by 8.69% and 3.27% for Cheras and Batu Muda, respectively. The significant improvement of model performance demonstrates that the improved method of EEMD has successfully increased the forecasting accuracy of LSTM compared to EMD. Moreover, white noises added in EEMD is remarkably efficient in extracting complex characteristic of the input sequence to successfully increase the performance and calculation time of the forecasting model.

On the other hand, the hybrid models of EEMD-LSTM and EMD-LSTM outperform individual LSTM in air quality forecasting at both monitoring stations. It can be observed that the decomposition method based on empirical mode has effectively improved the forecasting accuracy of LSTM. In this study, EEMD based model improved the performance of individual LSTM for Cheras dataset by 59.22%, 57.67%, 47.95% and 15.55% in terms of RMSE, MAE, MAPE and R^2^, respectively. Meanwhile, for Batu Muda dataset, EEMD improves the RMSE, MAE, MAPE and R^2^ of LSTM by 52.49%, 55.31%, 56.88% and 13.26%, respectively. The large percentage of improvement illustrates that the proposed decomposition method has greatly enhanced the forecasting procedure of LSTM and yielded accurate forecasting of PM2.5 concentration. Besides that, proposed EEMD-LSTM yield superior performance among the ensemble models of EMD-LSTM and EMD-GRU for both air quality datasets. Comparing the performance of EMD based models, it is found that EMD-GRU significantly outperforms EMD-LSTM for PM2.5 forecasting for both air quality monitoring stations. GRU is viewed as simplification of LSTM with fewer gate units in the architecture, shows better performance in air pollutant forecasting with the combination of data decomposition method. The superior performance of GRU also can be observed through the results based on the individual model at Cheras station. However, GRU performs poorer compared to LSTM for Batu Muda dataset. It can be perceived that the performance of both models depends on the distribution of training datasets and respective experiments^38^.

Based on the performance table, it is also found that the bidirectional architecture of LSTM yielded higher performance accuracy as compared to the general individual deep learning models namely LSTM and GRU, at both air quality monitoring stations. Comparing the performance of BiLSTM at both monitoring stations, it is found that the model improved LSTM performance errors at most by 4.3% in RMSE, 1.57% in MAE and 7.81% in MAPE. The model also improves the forecasting performance of GRU by 17.35%, 19.33% and 3.11% at most for RMSE, MAE and MAPE, respectively. The results illustrate that the improved architecture of the forward and backward layers in BiLSTM has positively impacted forecasting accuracy. The model is proven to be an efficient technique in forecasting and solving sequence datasets at both air quality monitoring stations with reliable performance accuracy. On the other hand, encoder-decoder architecture of seq2seq LSTM and CNN-LSTM perform poorer than other deep learning models. CNN-LSTM performs the worst among other deep learning models, with the highest performance error of 12.0066 μg/m^3^ for RMSE, 7.4250 μg/m^3^ for MAE, 38.48% for MAPE and the lowest R^2^ of 0.792 for PM2.5 forecasting at Cheras station. Similarly, for the Batu Muda dataset, CNN-LSTM yields the lowest performance accuracy compared to other deep learning models. This condition demonstrated that the architecture is less suitable for solving forecasting problems based on the sequence data at both monitoring stations.

### Multistep ahead forecasting

The proposed EEMD-LSTM is implemented to further analysed multistep ahead forecasting for both investigated monitoring stations. The multistep strategy used in this study is called direct strategy, where the proposed model is independently developed for each time horizon to forecast air pollutant concentration^39^. Table 6 presents the performance evaluation of EEMD-LSTM in forecasting PM2.5 concentration at 1 to 6-h of time horizon. The evaluation results depict decreasing forecasting performance as the time horizon increases at both locations. However, the performance of the proposed model is reliable, where the model yields the accuracy of R^2^ more than 90% at 5 h and 4 h time horizon for Cheras and Batu Muda stations, respectively. Therefore, examining the adequate combination of historical input and forecasting time horizon as well as spatial–temporal relationship would be effective in achieving optimum results for longer forecasting horizons.

**Table 6:** Multistep ahead forecasting of EEMD-LSTM.

		Time horizon (hour)					
		1	2	3	4	5	6
Cheras	RMSE (µg/m^3^)	4.2083	6.1535	6.7776	7.8380	7.8909	8.8216
	MAE (µg/m^3^)	2.8190	4.1204	4.7095	5.3166	5.2580	6.0334
	MAPE (%)	14.152	23.494	29.944	30.336	30.819	37.465
	R^2^	0.9780	0.9455	0.9339	0.9116	0.9104	0.8880
Batu Muda	RMSE (µg/m^3^)	4.8949	6.2990	6.9755	8.0898	8.8535	9.8561
	MAE (µg/m^3^)	2.7724	4.0043	4.5064	5.1294	5.7807	6.4409
	MAPE (%)	14.642	24.157	24.111	29.520	36.927	37.631
	R^2^	0.9673	0.9455	0.9332	0.9101	0.8923	0.8666

## Conclusion

In this study, an ensemble model of EEMD-LSTM is proposed to forecast 1-h ahead PM2.5 concentration at two air quality monitoring stations in an urban area. Considering the nonlinear and complex time-series data, the EEMD is firstly implemented to decompose sequence data of PM2.5 concentration into multiple simple features of intrinsic mode functions (IMFs). Then, LSTM is applied in mapping other air pollutant parameters and IMF values to establish an ensemble model for successive forecasting. Finally, the forecasted values of all modes are integrated to obtain the final forecasting results. The proposed hybrid model is applied based on two datasets of different air quality monitoring stations in order to validate its effectiveness under different pollution levels. A comparative analysis is conducted using four statistical evaluations to compare the proposed EEMD-LSTM model with other deep learning models for both monitoring stations. It is found that the performance of the proposed ensemble model yields outstanding performance and outweighs other deep learning models. Also, hybrid deep learning models based on the decomposition method have greatly improved the performance of individual models. Besides that, the results demonstrate that EEMD-LSTM has successfully learned and forecasted the PM2.5 concentration based on different dataset features. On the other hand, this study can be extended to forecast air pollutants by considering the effect of meteorology parameters in the vicinity of the study. The development of the hybrid forecasting model using an optimization method in selecting the optimum hyperparameters for the deep learning model is also suggested for future study improvement.

## Data Availability

The datasets used during the current study are available from the corresponding author on reasonable request.
